# Age-related accumulation of advanced oxidation protein products promotes osteoclastogenesis through disruption of redox homeostasis

**DOI:** 10.1038/s41419-021-04441-w

**Published:** 2021-12-14

**Authors:** Jingshen Zhuang, Xuebing Chen, Guixing Cai, Dizheng Wu, Chen Tu, Siyuan Zhu, Yusheng Huang, Ping Xu, Zhaoming Zhong

**Affiliations:** 1 Division of Spine Surgery, Department of Orthopaedics, Nanfang Hospital, Southern Medical University, Guangzhou, China; 2 Department of Forensic Medicine, School of Forensic Medicine, Southern Medical University, Guangzhou, China

**Keywords:** Senescence, Osteoporosis

## Abstract

Enhanced osteoclastogenesis is one of the major causes of age-related bone loss. Aging is accompanied by accumulation of advanced oxidation protein products (AOPPs). However, whether AOPPs accumulation contributing to the osteoclastogenesis with aging remains unclear. Here, we showed that AOPPs accumulation was associated with the enhanced osteoclastogenesis and deterioration of bone microstructure in aged mice. In vitro, AOPPs directly induced osteoclastogenesis by interaction with receptor activator of nuclear factor κ B (RANK) and the receptor for advanced glycation end products (RAGE) in the primary bone marrow monocytes. Bindings of AOPPs to RANK and RAGE were able to activate nicotinamide adenine dinucleotide phosphate (NADPH) oxidase, trigger generation of reactive oxygen species, then induce phosphorylation of mitogen-activated protein kinases and c-fos, upregulation of the nuclear factor of activated T cell c1, eventually induce bone marrow monocytes to differentiate into mature osteoclasts. Chronic exposure to AOPPs enhanced osteoclastogenesis and bone loss in mice, which could be alleviated by NADPH oxidase inhibitor apocynin. Local injection of AOPPs into subperiosteal area induced bone resorption at the site of administration, which was similar to the effect of RANK ligand. Together, these results suggested that AOPPs could serve as a novel regulator of osteoclastogenesis and AOPPs accumulation might play an important role in the development of age-related bone loss.

## Introduction

Maintenance of bone homeostasis is dependent on the balance between bone formation by osteoblasts and resorption by osteoclasts. Disruption to this balance may cause a sustained loss of bone mass and deterioration of bone microstructure in aging process [1]. Although the etiology of age-related bone loss is not well understood, it has been shown that the development of bone loss may be due to, in part, increased osteoclast formation or osteoclastogenesis with aging [2–6]. Receptor activator of nuclear factor κ B (RANK) and its ligand RANKL are classic molecules involved in osteoclastogenesis [7]. In addition, increasing evidences have showed that RANKL can also be substituted by other molecules during osteoclastogenesis, such as tumor necrosis factor-α [8, 9], lipopolysaccharide [10], interleukin-7 (ref. [11]), interleukin-11 (refs. [12, 13]), tumor necrosis factor superfamily member 14 (ref. [14]), transforming growth factor-β [15], leukotriene B4 (ref. [16]), secreted osteoclastogenic factor of activated T cells [17], a proliferation inducing ligand, B cell activating factor belonging to the tumor necrosis factor family, nerve growth factor, insulin-like growth factor I (IGF-I) and IGF-II [18]. Therefore, RANKL-dependent and RANKL-independent osteoclastogenesis may contribute to age-related bone loss.

Advanced oxidation protein products (AOPPs), which form by the reaction between chlorinated oxidants (HOCl/OCl^−^) and proteins, are defined as dityrosine-containing cross-linked proteins and serve as a novel marker of oxidative stress [19]. Plasma level of AOPPs is significantly higher in elderly people in comparison with adults and children/adolescents [20]. AOPPs can induce reactive oxygen species (ROS) generation and redox imbalance through activating nicotinamide adenine dinucleotide phosphate (NADPH) oxidases [21]. Our previous study and others have indicated that interaction of AOPPs with RAGE is involved in the regulation of diverse cellular functions, such as proliferation [22], differentiation [23], apoptosis [24], autophagy [25], and epithelial–mesenchymal transition [26]. We have also demonstrated that AOPPs accumulation was associated with aging and age-related bone loss [27]. AOPPs challenge aggravated osteoblast apoptosis and bone microstructure deterioration in aged rats [21, 28]. However, whether AOPPs accumulation contributes to the enhanced osteoclastogenesis with aging remains unknown.

In this study, we investigated the relationship between AOPPs and the osteoclastogenesis in age-related bone loss through the in vivo and in vitro model. AOPPs exposure induced osteoclastogenesis by binding of RANK and RAGE, and activating the downstream signaling. These findings suggested AOPPs accumulation contributes to enhanced osteoclastogenesis and age-related bone loss through disruption of redox homeostasis.

## Materials and methods

### AOPPs preparation and determination

AOPPs were prepared as described previously [21]. Briefly, 20 mg/ml rat serum albumin (RSA, Sigma-Aldrich, USA) was incubated with 40 mM hypochlorous acid (Fluke, Switzerland) for 30 min at room temperature. Free hypochlorous acid was removed by overnight dialysis against phosphate-buffered saline (PBS, pH 7.4) at 4 °C. All samples were passed through a Detoxi-Gel column (MA, USA) to remove contaminated endotoxin. AOPPs was then quantified as described with minor modifications [29].

### Animal experiments

Male animals were used to avoid the effects of estrogen deficiency on bone mass [30]. The 3-month-old and 18-month-old male C57/BL6 mice were obtained from Jinan Pengyue Experimental Animal Breeding Center (Shandong, China) and housed in the Southern Medical University Animal Experiment Center (Guangzhou, China). All animal experiment were approved by the Committee on Animal Experimentation and the Laboratory Animal Care and Use Committee of Southern Medical University. Plasma and tibias tissues were collected for the following analyses: (1) Plasma concentration of AOPPs were measured as described previously [29]; (2) Plasma levels of tartrate-resistant acid phosphatase (TRAP), C-telopeptide of type 1 collagen (CTX), Type 1 collagen amino-terminal (NTX) were detected by using the Enzyme-linked immunosorbent assay (ELISA) kits from Cusabio (China); (3) Left tibias sections were stained for TRAP with acid phosphatase kit (Sigma-Aldrich, USA) to label osteoclasts. (4) Bone microstructure of the right tibias were analyzed using micro-computed tomography (μCT) system (μCT80, Scanco Medical, Switzerland) as described previously [28].

Twenty-four male 3-month-old C57/BL6 mice were received the following treatments: daily intraperitoneal injection of PBS, unmodified MSA (50 mg/kg) and AOPPs (50 mg/kg), respectively. And another group was daily intraperitoneal injection of AOPPs (50 mg/kg) together with apocynin (NADPH oxidase inhibitor) at 100 mg/kg in drinking water. At the end of 16 weeks, the left tibias were collected for further study.

### Cells isolation, culture, and treatments

Primary bone marrow monocytes (BMMs) were prepared from adult male Sprague–Dawley rats and C57/BL6 mice as described previously [31]. BMMs were then treated with AOPPs (100 μg/ml), unmodified RSA (100 μg/ml). The cells treated with RANKL (100 ng/ml) were set as a positive group. For some experiments, BMMs were transfected with RANK or RAGE adenovirus (HanBio, China) for 36 h to knockdown of RAGE or RANK, the successful knockdown cells were used for subsequent experiments. In other experiments, BMMs were pretreated with some inhibitors, such as NADPH oxidase inhibitor apocynin at 100 μM, radicals scavenger superoxide dismutase (SOD) at 50 U/ml, JNK inhibitor SP600125 at 10 μM, p38 inhibitor SB203580 at 10 μM, and ERK1/2 inhibitor U0126 at 10 μM for 1 h, and followed by treatment with AOPPs.

### Osteoclastogenesis assay

BMMs were treated as described above. After 6 days, the culture supernatant was collected for detected TRAP activity by using TRAP assay Kit (Beyotime Biotech, China). Adherent cells were then performed TRAP staining according to the instructions.

### F-actin ring formation assay

BMMs were treated as described above. After 6 days, they were performed F-actin staining according to the instructions, the cells were counterstained with 4,6-diamidino-2-phenylindole (DAPI, Abcam, UK). F-actin ring was visualized with a confocal fluorescence microscopy (Olympus, Japan).

### Bone resorption assay

In vitro, BMMs were seeded on bovine cortical bone slice (6 × 6 mm size and 0.2 mm thickness), and were treated as described above. After 6 days, adherent cells were removed and the bone slices were scanning electron microscopy (Hitachi S-3000N, Japan) for observe the bone resorption pits.

In vivo, bone resorption model was established as previously described [32] with minor modifications. Twenty-four 8-weeks-old male Sprague–Dawley were injected subperiosteally over bilateral parietal bone every other day with AOPPs (10 µg/site), unmodified RSA (10 µg/site), PBS (100 µl/site) or RANKL (100 ng/site, R&D, USA). After 11 days, the parietal bone were collected and scanned with μCT.

### Surface plasmon resonance (SPR) analysis

The binding affinity of AOPPs for RANK or RAGE was measured by SPR analysis using a PlexArray HT A100 system (Plexera, USA). RANK and RAGE peptide (Sino biological, China) were diluted to 5 μg/ml in 10 mM sodium acetate buffer, pH 5.5. They were then immobilized on the sensor chip according to the manufacturer’s recommendations. Diluted AOPPs at concentrations ranging from 350 to 1050 μg/ml were injected, and 1050 μg/ml RSA was used as vehicle. The affinity assay was examined at 25 °C at a flow rate of 30 μl/min. AOPPs bindings to RANK or RAGE was recorded in real time. The *K*_D_, a value of the affinity between the two molecules, was calculated using the Plexera evaluation software.

### Immunofluorescent staining

BMMs were incubated with 100 μg/ml TRITC-labeled AOPPs (Taopu Biotech., China) for 20 min. The cells subsequently were incubated with RANK (sc-59981 Santa Cruz, USA) or RAGE (sc-365154 Santa Cruz, USA) antibodies and conjunct with FITC fluorescent antibody (ab6785, Abcam, USA), DAPI label the nucleus and obtained by the confocal fluorescence microscopy (Olympus, Japan).

### Flow cytometric analysis

BMMs were transfected with RANK or RAGE knock down adenovirus (HanBio, China) for 36 h, and human embryonic kidney (HEK) 293T cells were transfected with pHBAd-RANK, pHBAd-RAGE or pHBAd vector plasmids for overexpression by Lipofectamine 3000 Reagent (Thermofisher scientific, USA) for 48 h. The transfected cells were subsequently incubated with 100 μg/ml of TRITC-labeled AOPPs (Taopu Biotech., China) for 20 min, and the cells were subjected to fluorescence-activated cell sorting (FACS) analysis (BD, USA).

### Determination of intracellular ROS generation

Intracellular ROS was detected by the probe 2′,7′-dichlorofluorescein diacetate (DCFH-DA) as described in our previous study [21]. In brief, BMMs were incubated with 10 μM DCFH-DA for 20 min and then treated as described above. Fluorescence intensity (Ex/Em = 488/525) was measured on a SpectraMax M5 system (Molecular Devices, USA).

### Real-time PCR

Total RNA from cells was extracted using TRIzol reagent (Takara, Japan), and 1 μg of total RNA was reverse transcribed by using a high-capacity cDNA kit (Takara, Japan). cDNA were used as templates for real-time polymerase chain reaction (RT-qPCR). Relative expression was calculated using the ΔΔ^ct^ method. The primers sequences were shown in the supplement material table 1.

### Western blot analysis

Protein expression was analyzed by western blot using specific antibodies as described previously [21]. Antibodies against p38 (#8690), phosphor-p38 (#4511), ERK1/2 (#4695), phosphor-ERK1/2 (#4370), JNK (#9252), phosphor-JNK (#4668), c-fos (#4384), phosphor-c-fos (#5348), nuclear factor of activated T-cells (NFATc1, #8032), and GAPDH (#5174) were from Cell Signaling Technology (Beverly, USA); Antibodies against p47^phox^ (ab795), p22^phox^ (ab75941), NOX1 (ab131088), NOX4 (ab133303) were from Abcam (UK). Immunoreactive proteins were detected with horseradish peroxidase (HRP)-coupled secondary antibodies (goat anti rabbit, ab6721; rabbit anti goat, ab6741, Abcam, UK).

### Immunoprecipitation

The interaction of AOPPs with RANK and RAGE, p47^phox^ with p22^phox^, Nox2 and Nox4, and phosphorylated p47^phox^ in cultured BMMs were determined by immunoprecipitation as described previously [21]. Antibody against AOPPs was kindly gifted from Dr. Jianwei Tian [33] (Southern Medical University, China). Antibody against RANK (sc-59981), RAGE (sc-365154) were from Santa Cruz (CA, USA). Antibodies against p47^phox^ (ab795), phosphor-p47^phox^ (ab166930), p22^phox^ (ab75941), NOX1 (ab131088), NOX4 (ab133303) were from Abcam (UK). Immune complexes were then tested by western blotting.

### Statistical analysis

All the experiments were repeated at least three times. Continuous variables were presented as mean ± Standard Deviation (SD). One-way ANOVA analysis was used to detect differences among groups. Two-tailed *P*-value of less than 0.05 was considered statistically significant. Statistical analysis was conducted with SPSS 20.0 software (SPSS Inc, Chicago, IL).

## Results

### AOPPs accumulation was associated with enhanced osteoclastogenesis in aged mice

**Fig. 1:**
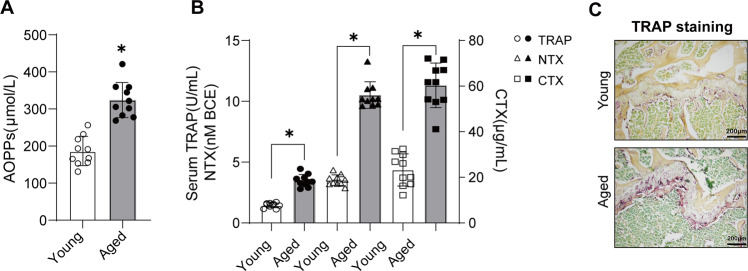
AOPPs accumulation is associated with the enhanced osteoclastogenesis in aged mice. **A** Serum AOPPs level significantly increased in aged mice (18-month-old) compared with the young mice (3-month-old). **B** The serum bone resorption markers, such as TRAP (left *Y* axis), NTX (left *Y* axis) and CTX (right *Y* axis), were significantly increased in aged mice. **C** TRAP staining of tibia showed the increase of TRAP positive cells in aged mice. Data were presented as mean ± SD. *p < 0.05 versus the young group.

To address correlation between AOPPs accumulation and age-related bone loss, the serum level of AOPPs, bone resorption markers and bone microstructure were analyzed. The serum level of AOPPs was significant elevated in aged group compared with young group (Fig. 1A). As shown in Fig. 1B, the bone resorption markers, such as TRAP activity, NTX and CTX, were increased in the aged group. An obvious increase of TRAP-positive cells was observed in aged group (Fig. 1C). Furthermore, the reduced BMD and deteriorated bone microstructure were observed in the aged group (Supplement Fig. 1).

### AOPPs directly induced osteoclastogenesis in vitro

**Fig. 2:**
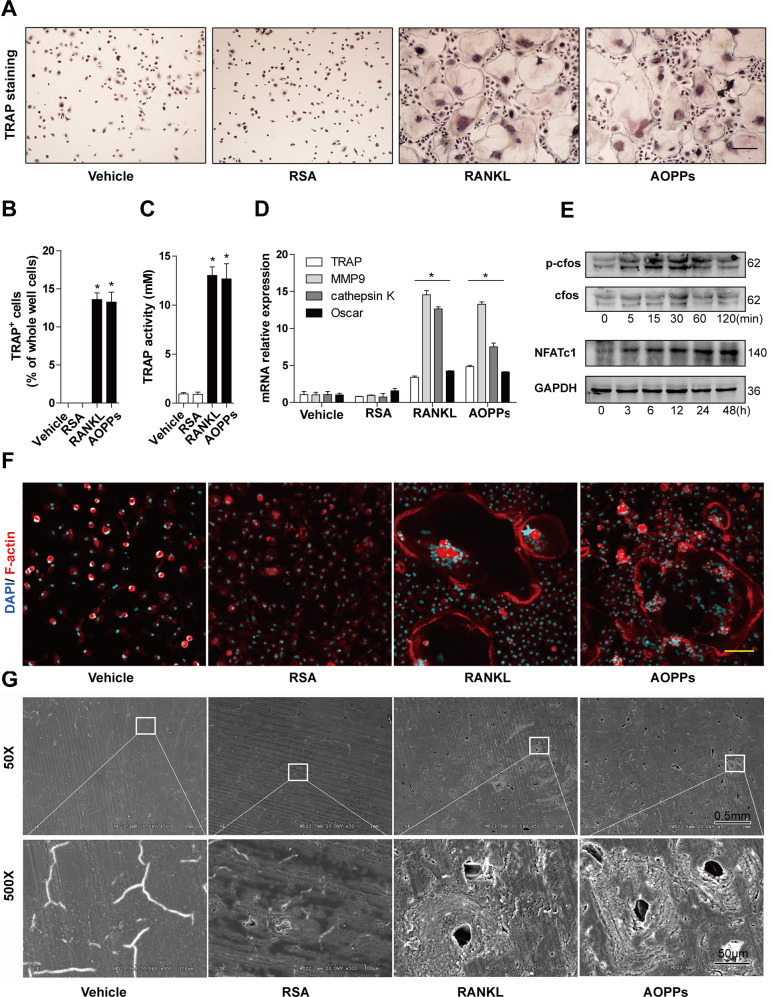
AOPPs directly induced osteoclastogenesis in vitro. Rat BMMs were stimulated with or without AOPPs for 6 days, RANKL-treated cells were used as positive control, and they were not treated with MCSF. **A**, **B** AOPPs treatment induced the formation of TRAP-positive cells. Scale bar = 200 μm. **C** AOPPs treatment significantly increased TRAP activity, **D** AOPPs treatment induced gene expression of osteoclast differentiation markers, such as TRAP, MMP9, cathepsin K and Oscar. **E** AOPPs treatment significantly increased the phosphorylated c-fos and expression of NFATc1. **F** AOPPs treatment induced the formation of F-actin ring, scale bar = 100 μm. **G** Obvious resorption pits on bone slice formed in the AOPPs group and RANKL group, but not vehicle and RSA group. Data were presented as mean ± SD. *p < 0.05 versus the vehicle-treated group.

We next examined the effect of AOPPs on osteoclastogenesis in vitro. AOPPs treatment significantly induced the formation of TRAP positive multinuclear cells (Fig. 2A, B) and increased TRAP activity (Fig. 2C) in the rat BMMs. Increased gene expression of osteoclast differentiation markers, such as TRAP, matrix metallopeptidase 9 (MMP9), cathepsin K and oscar, were shown in the AOPPs-treated group, but not in the RSA and vehicle-treated groups (Fig. 2D). The osteoclast differentiation in the AOPPs-treated BMMs was similar to that in the RANKL-treated rat BMMs (Fig. 2A–D). The transcription factors c-fos and NFATc1 are critical components for osteoclastogenesis [34, 35]. AOPPs exposure also induced the phosphorylation of c-fos and increase of NFATc1 expression (Fig. 2E). Bone degradation by osteoclasts depends on the formation of a sealing zone, which is a ring-like F-actin-rich structure. In present study, the formation of F-actin ring was observed in the AOPPs-treated BMMs measured by confocal microscopy, which was similar to RANKL-treated cells (Fig. 2F). When rat BMMs were cultured on bone slice in the presence of AOPPs or RANKL treatment, the obvious resorption pits on bone slice were observed by the electron microscopy (Fig. 2G). Furthermore, RANKL wasn’t detected in the culture supernatants of AOPPs-treated cells by ELISA, which ruled out autocrine production of RANKL (Supplement Fig. 2A). To confirm the induce osteoclastogenesis of AOPPs in mice BMMs, we examined the effect of AOPPs in mice BMMs and the similar effect were shown in Supplement Fig. 3.

### Binding of AOPPs to RANK and RAGE

**Fig. 3:**
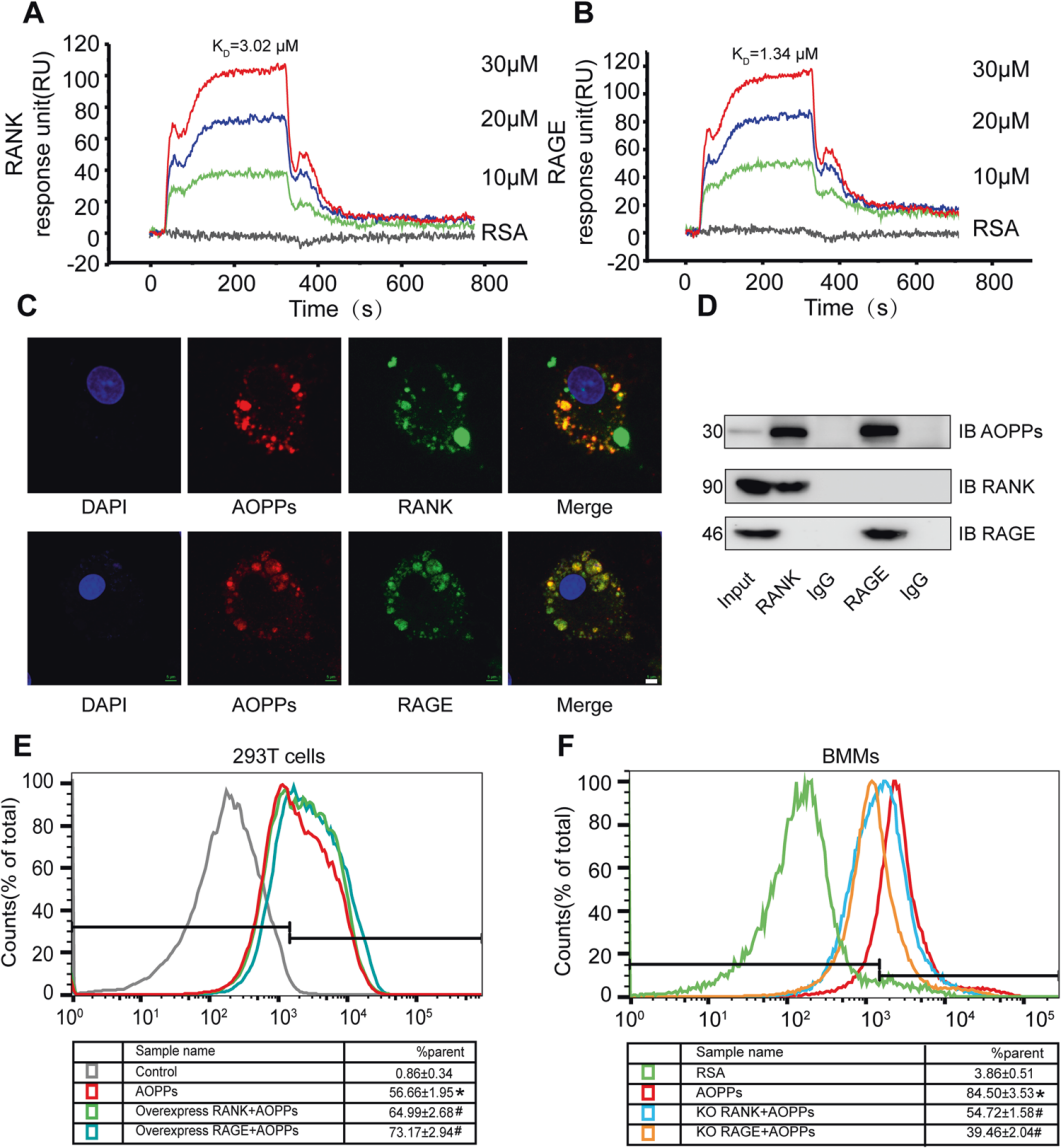
Binding of AOPPs to RANK and RAGE. **A**, **B** SPR binding-affinity showed AOPPs bound to RANK and RAGE. **C** Immunofluorescence staining showed that TRITC-AOPPs (red) co-localized with RANK (green, up) or RAGE (green, down) on the plasma membrane of BMMs. Scale bars = 5 µm. **D** BMMs were treated with AOPPs. Co-immunoprecipitation (IP) analysis of AOPPs and RANK or RAGE association in BMMs. Immunoblot (IB) probed using indicated antibodies. **E** FACS analysis of AOPPs binding to HEK293T cells transfected with RANK or RAGE. RANK increased from 56.66 ± 1.95% (red, positive cells) to 64.99 ± 2.68% (green, RANK overexpressing cells) and 73.17 ± 2.94% (blue, RAGE overexpressing cells), and the control cell (gray) was 0.86 ± 0.34%. **F** In BMMs, knockdown of endogenous RANK decreased TRITC-AOPPs binding from 84.50 ± 3.53% (red) to 54.72 ± 1.58% (blue, RANK knockdown cells), and 39.46 ± 2.04% (orange, RAGE knockdown cells). *p < 0.05 versus the vehicle-treated group. ^#^*p* < 0.05 versus the AOPPs-treated group.

To test whether AOPPs directly interacted with RANK, an essential signaling receptor for osteoclast differentiation, and RAGE, a known receptor for AOPPs, four different approaches were employed. First, SPR analyses showed that AOPPs bound to RANK with a *K*_D_ of 3.02 μM (Fig. 3A), and bound to RAGE with a *K*_D_ of 1.34 μΜ (Fig. 3B). But RSA didn’t bind to RANK or RAGE as the negative group. Second, BMMs were treated with TRITC-AOPPs. The result showed that TRITC-AOPPs could co-localize with RANK or RAGE on the plasma membrane of BMMs (Fig. 3C). The similar results were detected in HEK293T cells with overexpression of RANK or RAGE (Supplement Fig. 4). Third, HEK293T cells with overexpression of RANK or RAGE were treated with AOPPs. Co-immunoprecipitation and western blot showed that both the RANK and RAGE could directly bind with AOPPs (Fig. 3D). Finally, we quantified the ratio of AOPPs binding to RANK or RAGE in HEK293T cells. The ratio value of AOPPs binding to cells showed that overexpressed RANK (64.99%) and RAGE (73.17%), were higher than those in control HEK293T cells (0.86%) (Fig. 3E). Conversely, BMMs after knockdown of endogenous RANK (54.72%) or RAGE (39.46%) had lesser binding to AOPPs than those in control BMMs (84.50%) (Fig. 3F).

### AOPPs activated NADPH oxidase by RANK and RAGE signaling

**Fig. 4:**
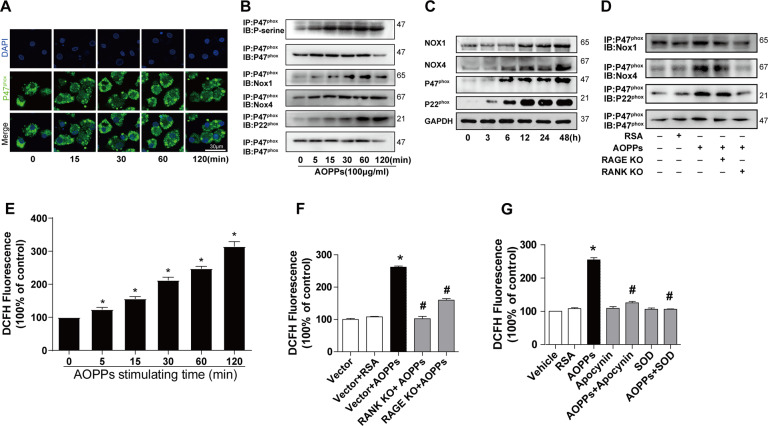
AOPPs activated NADPH oxidase and increased intracellular ROS generation by RANK and RAGE signaling. **A** BMMs were treated with AOPPs for 0, 15, 30, 60, 120 min, then p47^phox^ were measured by immunofluorescence staining. Representative confocal microscope images of AOPP-induced membrane translocation of p47^phox^. Scale bars = 30 µm. **B** Co-immunoprecipitation analysis showed that AOPP treatment enhanced the binding of p47^phox^ to Nox1, Nox4, and p22^phox^. **C** Western bolting analysis revealed that AOPPs treatment increased expression of NADPH oxidase subunits Nox1, Nox4, p22^phox^ and p47^phox^. **D** Co-immunoprecipitation analysis showed that the enhanced binding of p47^phox^ to Nox1, Nox4, and p22^phox^ induced by AOPPs were inhibited after knockdown of RANK or RAGE. **E** Intracellular ROS was detected by DCFH-DA. AOPPs treatment induced intracellular ROS generation. **F**, **G** AOPPs-induced ROS generation were significantly blocked after knockdown RANK or RAGE or pre-treatment with apocynin (100 μM, a NADPH oxidase inhibitor) or SOD (50 U/ml, a radical scavenger superoxide dismutase). Representative images of three view fields of per experiment were shown. Each co-IP was performed three times experiments and blotted separately each time. Data were presented as mean ± SD. *p < 0.05 versus the vehicle-treated group. ^#^*p* < 0.05 versus AOPPs-treated group.

Then, we analyzed the activation of NADPH oxidase in AOPPs-treated BMMs. As shown in Fig. 4A, B, AOPPs challenge resulted in membrane translocation of cytosolic subunit p47^phox^ (Fig. 4A) and the phosphorylation of NADPH oxidase subunits (Fig. 4B), which promoted the binding of p47^phox^ to the membrane subunits Nox1, Nox4, and p22^phox^ (Fig. 4B). Meanwhile, the expression of NADPH oxidase subunits, such as Nox1, Nox4, p22^phox^, and p47^phox^, were also upregulated after AOPPs treatment (Fig. 4C). Importantly, the knockdown of RANK or RAGE significantly inhibited the AOPP-induced binding of p47^phox^ to the membrane components (Fig. 4D).

### AOPPs increased intracellular ROS generation by activation of NADPH oxidase

Previous studies showed that AOPPs involved in cell differentiation by modulating intracellular ROS generation [36], and NADPH oxidase was one of the main sources of endogenous ROS [37]. Herein, we examined intracellular ROS levels in AOPPs-treated BMMs. As shown in Fig. 4E–G, AOPPs incubation increased ROS generation in a time-dependent manner (Fig. 4E). Furthermore, AOPP-induced ROS generation were significantly blocked after knockdown RANK or RAGE expression (Fig. 4F), or the pre-treatment of apocynin (inhibitor of NADPH oxidase) or superoxide dismutase (SOD, a free radical scavenger) (Fig. 4G).

### AOPPs activated MAPKs by ROS generation

**Fig. 5:**
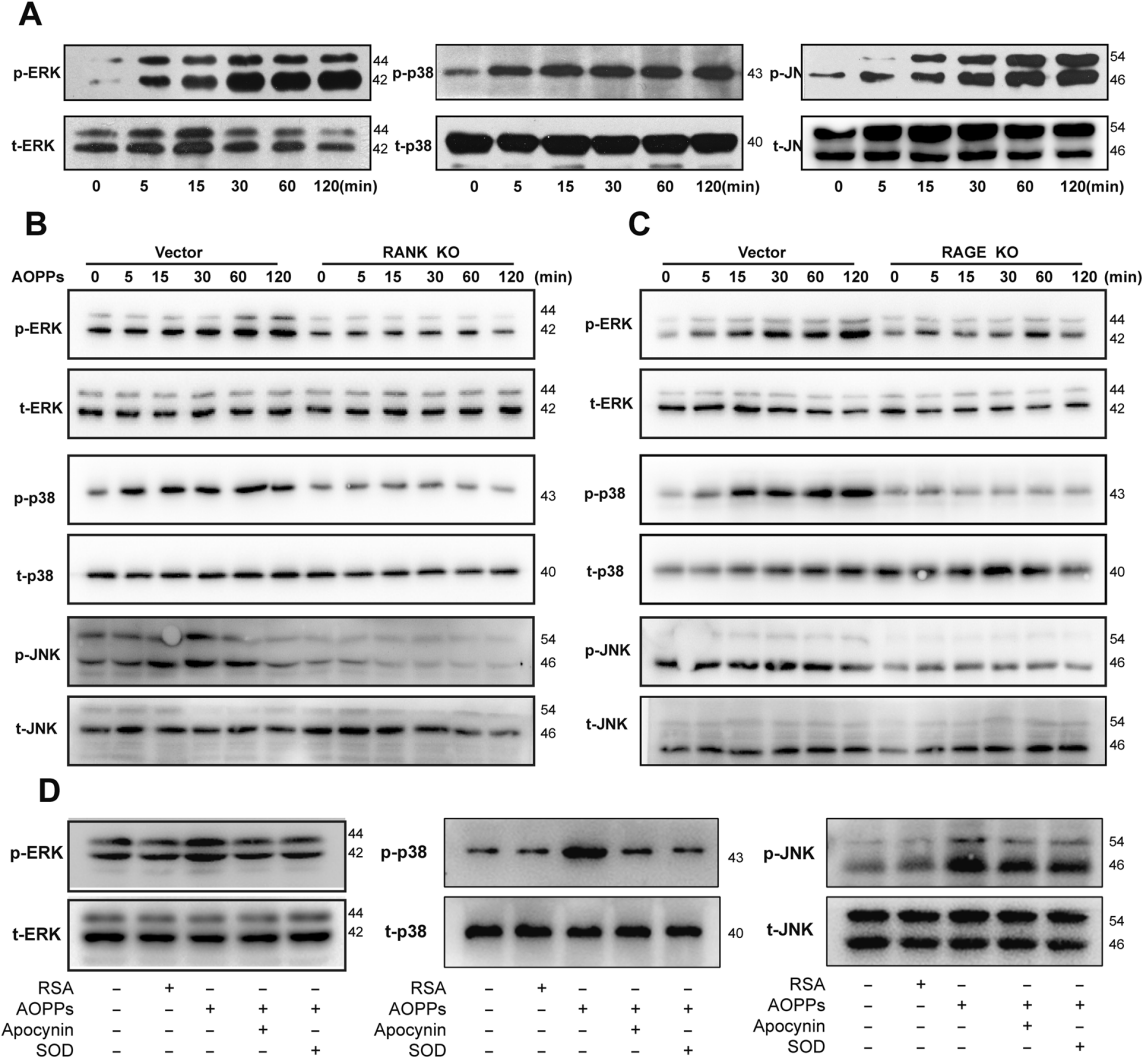
AOPPs activated MAPKs by ROS generation. **A** AOPPs treatment significantly increased phosphorylation of ERK1/2, p38, and JNK in BMMs. **B**, **C** Knockdown of RANK or RAGE was significantly blocked the AOPPs-induced phosphorylation of ERK1/2, p38, and JNK. **D** Pre-treatment of apocynin and SOD were significantly reverse the AOPPs-induced phosphorylation of ERK1/2, p38, and JNK.

Three major members of MAPKs, including ERK1/2, p38, and JNK, played pivotal roles in process of osteoclastogenesis and bone resorption [38]. Thus, we investigated whether they were activated during osteoclastogenesis induced by AOPPs. As shown in Fig. 5A, AOPPs treatment resulted in a markedly increase phosphorylation of ERK1/2 (left), p38 (middle), and JNK (right), which were significantly blocked after knockdown of RANK (Fig. 5B) or RAGE (Fig. 5C), or the pre-treatment of apocynin (an inhibitor of NADPH oxidase) or SOD (a free radical scavenger) (Fig. 5D).

### AOPPs induced osteoclastogenesis by the RANK and RAGE dependent redox signaling

**Fig. 6:**
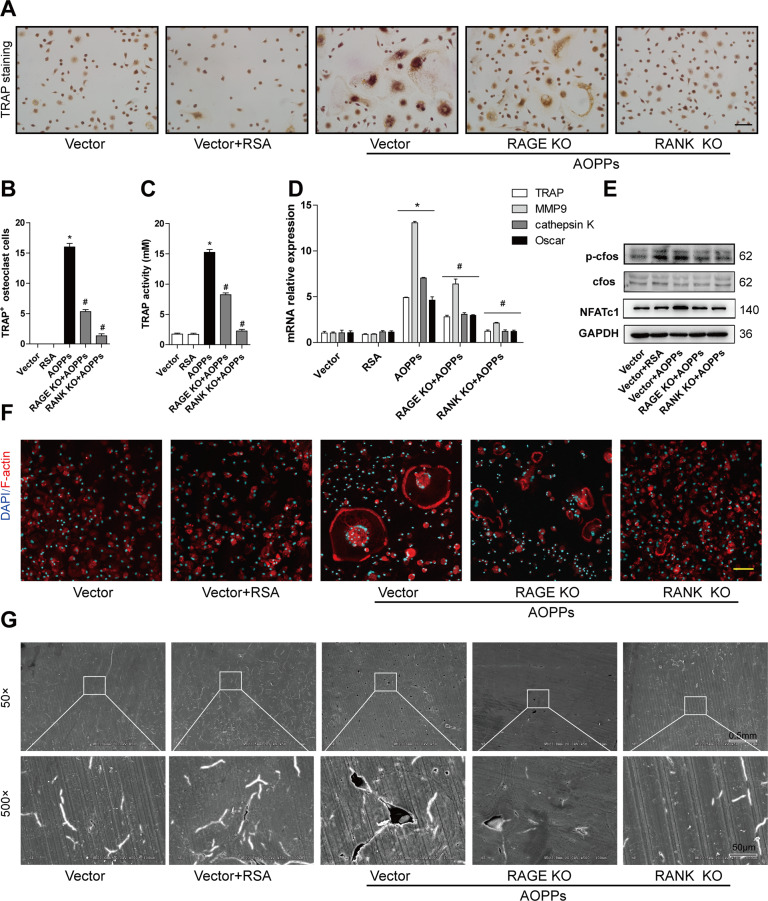
AOPPs induced osteoclastogenesis by RANK and RAGE signaling. **A**, **B** Knockdown of RANK or RAGE suppressed the formation of TRAP positive cells induced by AOPPs. Scale bars = 200 μm. **C** Knockdown of RANK or RAGE decreased the TRAP activity, **D** Knockdown of RANK or RAGE inhibited gene expression of TRAP, MMP9, cathepsin K, and Oscar. **E** Knockdown of RANK or RAGE inhibited the expression of NFATc1 and phosphorylation of c-fos, **F** Knockdown of RANK or RAGE inhibited the formation of F-actin rings induced by AOPPs. Scale bars = 100 μm. **G** Knockdown of RANK or RAGE blocked AOPPs-induced bone resorption. Data expressed as mean ± SD. *p < 0.05 versus the vetor group; ^#^*p* < 0.05 versus the AOPPs group.

To investigate whether AOPPs induced osteoclastogenesis by interacting with RANK or RAGE, BMMs were knocked out RANK or RAGE before AOPPs stimulation. Knockdown of RANK or RAGE significantly suppressed the AOPPs-induced form of TRAP positive cells (Fig. 6A, B) and increase of TRAP activity (Fig. 6C). Interestingly, RAGE knockdown only partially affected the form of TRAP positive cells, including decreased cell number and smaller cell size. The expression of genes TRAP, MMP9, cathepsin K, and Oscar induced by AOPPs were markedly decreased after knockdown of RANK or RAGE (Fig. 6D). The phosphorylation of c-fos and the expression of NFATc1 were inhibited after knockdown of RANK or RAGE (Fig. 6E). Knockdown of RANK or RAGE also decreased the formation of F-actin rings (Fig. 6F) and bone-resorption pits (Fig. 6G).

**Fig. 7:**
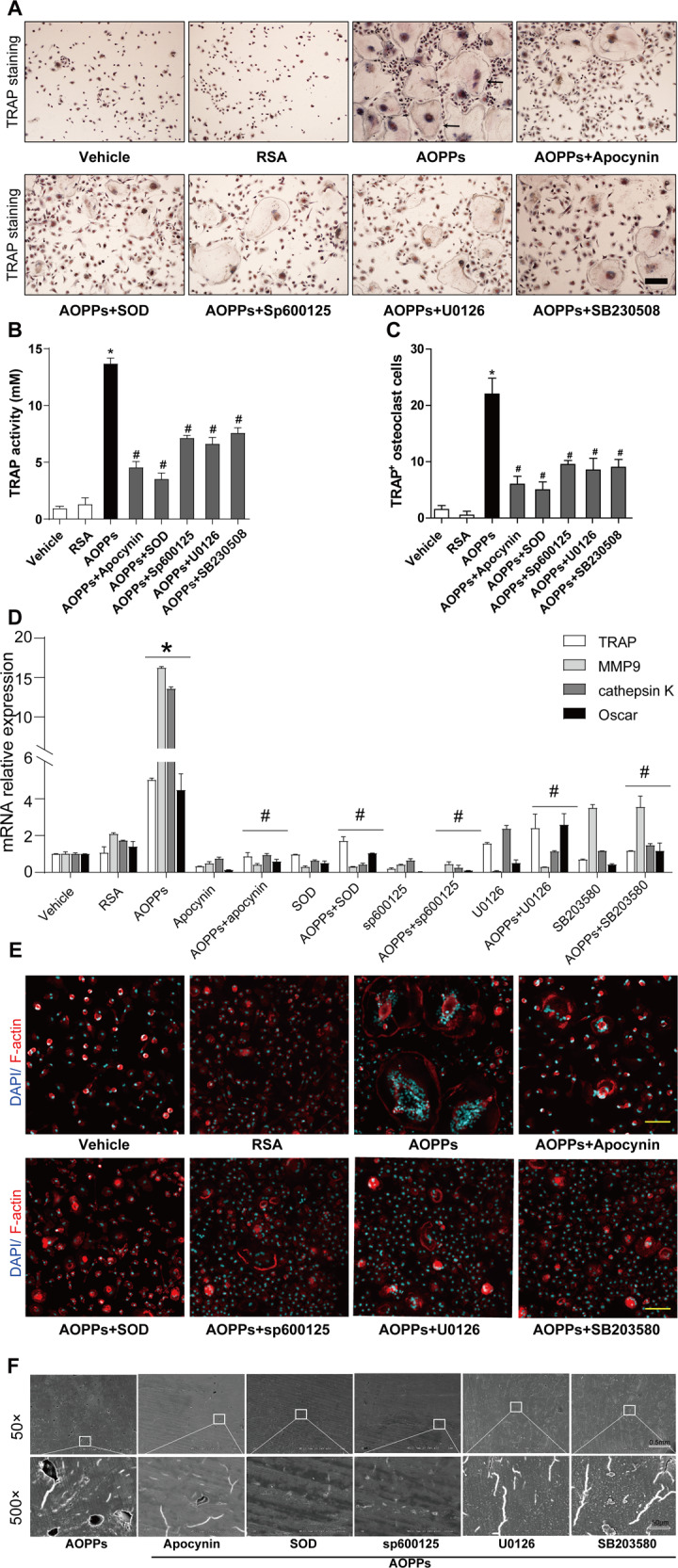
AOPPs induced osteoclastogenesis through the NADPH oxidase–mediated redox signaling. **A**, **B** Apocynin (NADPH oxidase inhibitor), SOD, SP600125 (JNK inhibitor), U0126 (ERK1/2 inhibitor), and SB20358 (p38 inhibitor) suppressed the formation of TRAP positive Osteoclast induced by AOPPs. Scale bars = 200 μm. **C** Apocynin, SOD, SP600125, U0126, and SB20358 decreased the TRAP activity. **D** Blocked of apocynin, SOD, SP600125, U0126, and SB20358 inhibited gene expression of TRAP, MMP9, cathepsin K, and Oscar. **E** Blocked of apocynin, SOD, SP600125, U0126, and SB20358 inhibited the formation of F-actin rings induced by AOPPs. Scale bars = 100 μm. **F** Apocynin, SOD, SP600125, U0126, and SB20358 blocked AOPPs-induced bone resorption. Data were presented as mean ± SD. *p < 0.05 versus the vehicle-treated group; ^#^*p* < 0.05 versus AOPPs group.

To clarify the role of NADPH oxidase-mediated redox sensitive signaling in osteoclastogenesis induced by AOPPs, BMMs cells were pre-incubated with some inhibitors of this signaling axis following AOPPs stimulation. AOPPs-induced form of TRAP positive cells (Fig. 7A, B), increase of TRAP activity (Fig. 7C), expression of TRAP, MMP9, cathepsin K, and Oscar gene (Fig. 7D) were significantly suppressed by NADPH oxidase inhibitor apocynin, ROS scavenger SOD, JNK inhibitor SP600125, ERK1/2 inhibitor U0126, and p38 inhibitor SB20358. Furthermore, the formation of F-actin rings (Fig. 7E) and bone-resorption pits (Fig. 7F) were also decreased after treatment of the above inhibitors.

### Chronic exposure to AOPPs induced osteoclastogenesis and bone loss in vivo

**Fig. 8:**
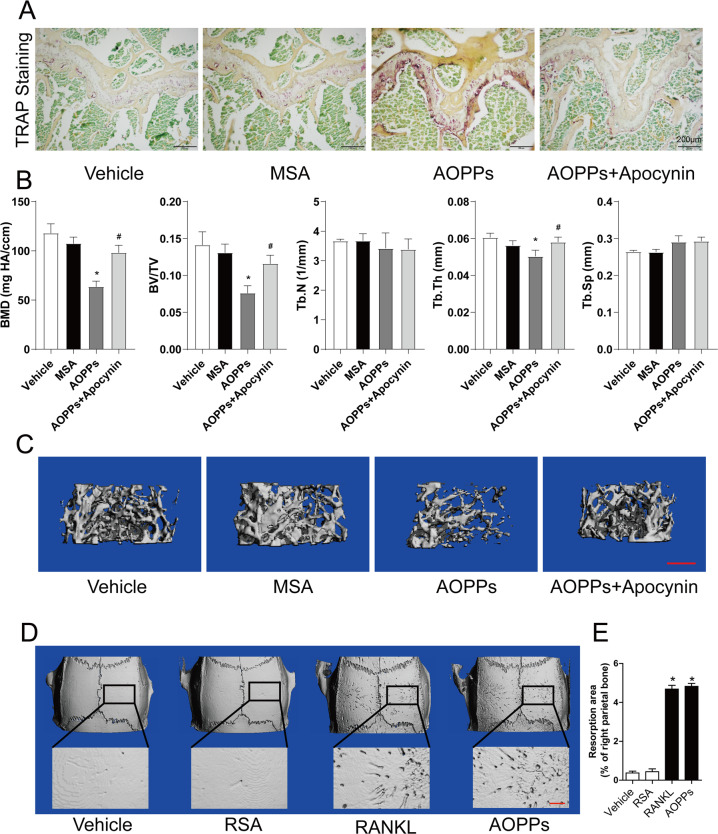
Chronic exposure to AOPPs induced osteoclastogenesis and bone loss in vivo. The 3-month-old mice were treated by daily intraperitoneal injection of AOPPs for 16 weeks with or without apocynin, the samples of proximal tibias were used for TRAP staining and μCT analyses. **A** Chronic loading of AOPPs induced the increase of TRAP positive cells, which could be inhibited by administration of apocynin. **B** AOPP challenge decreased the BMD (bone mineral density), BV/TV (Bone Volume/Total Volume) and d Tb.Th (Trabecular Thickness), but not Tb.N (Trabecular Number) and Tb.Sp (Trabecular Spacing), which could be alleviated by administration of apocynin. **C** Representative images of μCT three-dimensional reconstruction in proximal tibias. **D** Local AOPPs aggravated bone resorption in vivo. Sprague–Dawley rats were injected subperiosteally over bilateral parietal bone. Representative images of μCT three-dimensional reconstruction. **E** The resorption area was calculated by image J software. Scale bars = 1 mm. *p < 0.05 versus the vehicle-treated group; ^#^*p* < 0.05 versus AOPPs group.

Daily intraperitoneal injection of AOPPs in mice for 16 weeks and TRAP staining was conducted to examine whether AOPPs induced osteoclastogenesis in vivo. As shown in Fig. 8A, chronic loading of AOPPs induced the increase of TRAP positive cells in proximal tibias. We then evaluated the effect of AOPPs on bone microstructure and bone mass. The μCT evaluation showed that AOPPs challenge decreased the BMD and trabecular indexes (BV/TV, Tb. Th) in proximal tibias (Fig. 8B, C). These effects were alleviated by administration of apocynin, a NADPH oxidase inhibitor (Fig. 8A–C).

### Local injection of AOPPs induced bone resorption in vivo

To further detect whether AOPPs induce osteoclastogenesis, we examined the effect of local injection of AOPPs on bone resorption. As shown in Fig. 8D, E, the local injection of AOPPs into subperiosteal area resulted in obvious bone resorption in the cranial bone of rats. Interestingly, bone resorption induced by AOPPs was similar to the effect of RANKL.

## Discussion

AOPPs is the biomarker of oxidative damage to protein and involved in aging process and the development of some age-related diseases [22, 24, 39–41]. Level of AOPPs in plasma and bone tissue increased with aging and were negatively associated with BMD [27]. Reduction in bone formation by decreased recruitment of osteoblasts and elevation of bone resorption by enhanced activity of osteoclasts are the underlying mechanism of age-related bone loss [41]. We have shown that AOPPs can aggravate osteoblast apoptosis and bone microstructure deterioration in aged rats [21, 28]. In the present study, we certified that AOPPs had the potential to induce osteoclast differentiation and activity. AOPPs accumulation promoted osteoclastogenesis and may be actively involved in the process of age-related bone loss.

RANKL-RANK axis is essential for osteoclastogenesis. Binding of RANKL to RANK can activate a variety of downstream signaling pathways required for differentiation, function and survival of osteoclasts [7]. However, there is growing evidence to support the existence of RANKL-independent osteoclastogenesis [42]. In the present study, we demonstrated that the accumulation of AOPPs was correlated with change of BMD and serum bone resorption markers in aged mice. The subperiosteal injection of AOPPs resulted in bone resorption at the site of administration, which was similar to RANKL-induced bone resorption in vivo. In the absence of RANKL, we found that AOPPs efficiently induced BMMs fusion to TRAP-positive multinucleated cells, the formation of F-actin rings and bone-resorption pits in vitro. Therefore, our data show that AOPPs can mediate osteoclastogenesis independently of RANKL.

RANK is a type I membrane protein and expressed on the surface of osteoclast progenitor cells. Upon ligand binding, RANK can initiate osteoclastogenic signal transduction. It was reported that RANK-deficient mice displayed osteopetrosis due to a lack of osteoclasts [43]. RANKL is an important ligand of RANK, the binding of RANKL to RANK has be considered to be essential step for osteoclast formation and function [44]. In this study, we demonstrated that AOPPs was another ligand for RANK. Binding of AOPPs to RANK activated a cascade of intracellular signaling, which ultimately induced BMMs to differentiate into TRAP positive multinucleated cells with bone resorbing ability. Knockdown of RANK significantly suppressed the osteoclast formation and function. RAGE was widely reported to be a native receptor of AOPPs [45, 46], but knockdown of RAGE only partially attenuated osteoclast formation induced by AOPPs in our study. Therefore, RANK signaling plays a key role in AOPPs-induced osteoclastogenesis.

NADPH oxidase is one of the main sources of ROS generation [37]. ROS, including superoxide and hydrogen peroxide, are crucial components to regulate the process of osteoclastogenesis [47, 48]. NADPH oxidase Nox4^−/−^ mice display higher bone density, reduced the numbers and gene markers of osteoclasts [49]. It was demonstrated that AOPPs could activate the NADPH oxidase, derive ROS generation and disrupt redox homeostasis [26, 50, 51]. In the present study, we found that AOPPs promoted ROS generation via the activation of NADPH oxidase in vitro. Excessive ROS generation played important role in osteoclastogenesis induced by AOPPs, which was suppressed by treatment of apocynin (a NADPH oxidases inhibitor) or SOD (an oxidant scavenger). Furthermore, MAPK family members, including ERK1/2, JNK, and p38, are redox sensitive and serve as key players in the maintenance of bone homeostasis [52]. In this study, the activation of MAPKs family was involved in AOPPs-induced osteoclastogenesis. Thus, NADPH oxidase-mediated redox signaling play a key role in the process of AOPPs-induced osteoclastogenesis and bone resorption.

In conclusion, our data indicate that AOPPs accumulation is associated with aging and promotes osteoclastogenesis, which contributes to age-related bone loss. Thus, AOPPs can serve as a novel regulator of osteoclastogenesis and AOPPs accumulation might play an important role in the development of age-related bone loss. AOPPs are not only the oxidative stress biomarkers, but also the activators of ROS generation and oxidative stress. Reducing AOPP generation and its cascading effect may be block osteoclastogenesis and eventually helpful for treating age-elated bone loss.

## Supplementary information

supplementary legends

Supplemental figure1

Supplement figure 2

Supplemental figure3

Supplemental figure4

Supplement tabel 1

Title page

Author Contribution Form

A reproducibility checklist

## Data Availability

The data that support the findings of this study are available from the corresponding author upon reasonable request.
