# Supplementary material for: Age-related accumulation of advanced oxidation protein products promotes osteoclastogenesis through disruption of redox homeostasis
Source: Cell Death Dis. 2021 Dec 14;12(12):1160. doi: 10.1038/s41419-021-04441-w (PMC8671415; doi:10.1038/s41419-021-04441-w)
Supplement: Supplementary file 1 — supplementary legends [file 41419_2021_4441_MOESM1_ESM.docx]

**Supplement figure 1.** **Deterioration of bone microstructure in aged mice.** (A) μCT three-dimensional showed the deterioration bone microstructure in aged mice. (B)The bone mineral density (BMD), bone volume/total volume (BV/TV), trabecular number (Tb. N) and trabecular thickness (Tb. Th) were decrease, and trabecular spacing (Tb.Sp) were increased in the old group compared with the young group. Scale bars=0.5mm. * *p* <0.05 versus the young group.

**Supplement figure 2.** RANKL wasn’t detected by ELISA assay in the culture supernatants of AOPP-treated cells.

**Supplement figure 3.** **AOPPs directly induced osteoclastogenesis in mice BMMs.** Mice BMMs were stimulated with or without AOPPs for 6 days, RANKL-treated cells were used as positive control, and they were not treated with MCSF. (A) AOPPs treatment induced the formation of TRAP-positive cells. Scale bar =200μm. (B) The formation of F-actin ring was observed in the AOPPs-treated mice BMMs measured by confocal microscopy, which was similar to that in RANKL-treated cells. (C) Increased gene expression of osteoclast differentiation markers, such as TRAP, MMP9, cathepsin K and Oscar were shown in the AOPPs-treated group, but not in vehicle-treated groups. Data were presented as mean ± SD. * *p*<0.05 versus the vehicle-treated group.

**Supplement figure 4.** **Binding of AOPPs to RANK and RAGE.** HEK293T cells overexpress with RANK or RAGE were treated with TRITC-AOPPs. (A-B) AOPPs co-localized with RANK or RAGE on the plasma membrane of HEK293T cells, but not RSA. Representative images of RANK or RAGE (green), AOPPs or RSA (red) and DAPI (blue), Scale bars =50 µm.
