# Supplementary figures and images for: Age-related accumulation of advanced oxidation protein products promotes osteoclastogenesis through disruption of redox homeostasis
Source: Cell Death Dis. 2021 Dec 14;12(12):1160. doi: 10.1038/s41419-021-04441-w (PMC8671415; doi:10.1038/s41419-021-04441-w)

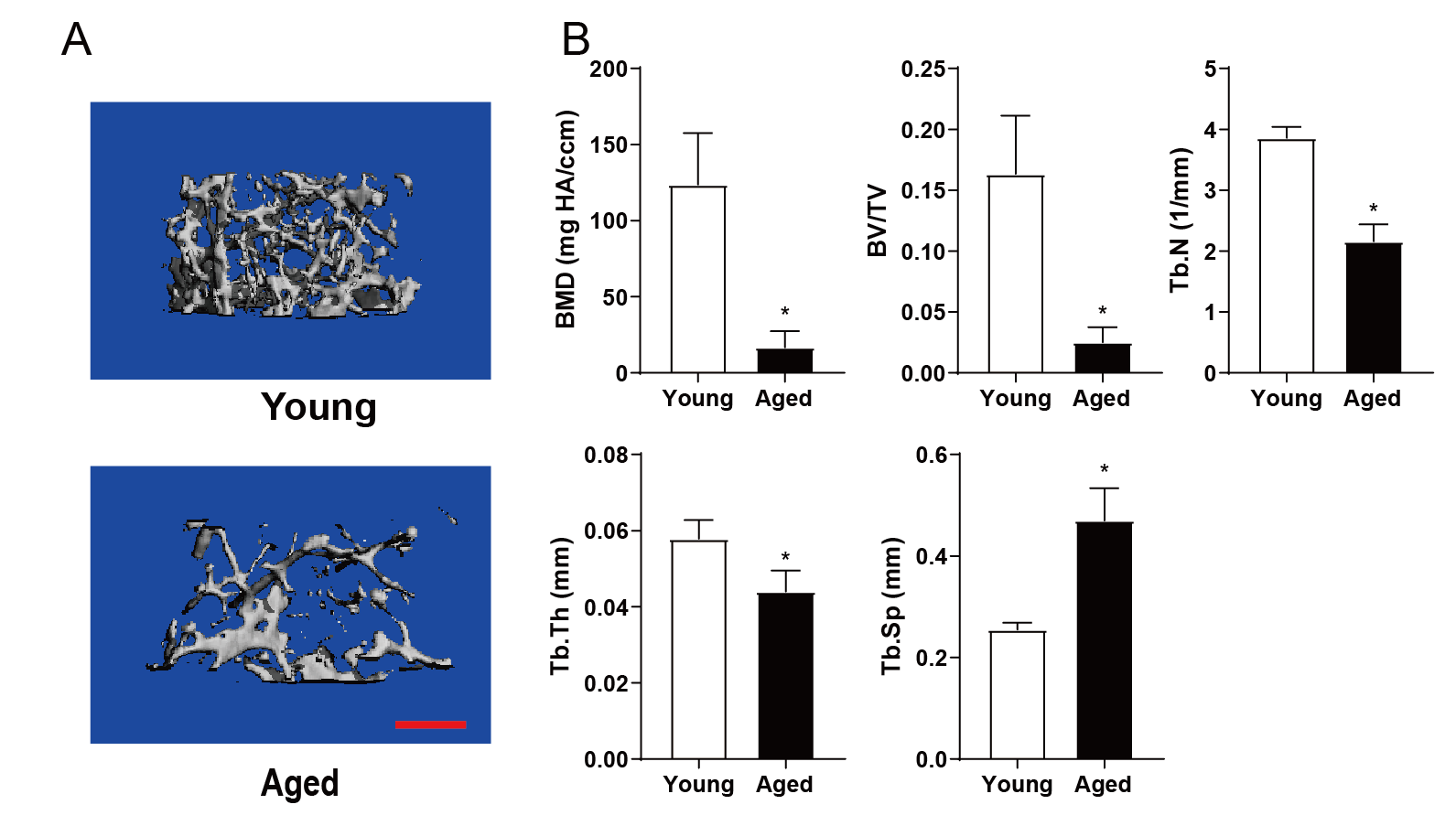

Supplement: Supplementary file 2 — Supplemental figure1 [file 41419_2021_4441_MOESM2_ESM.png]

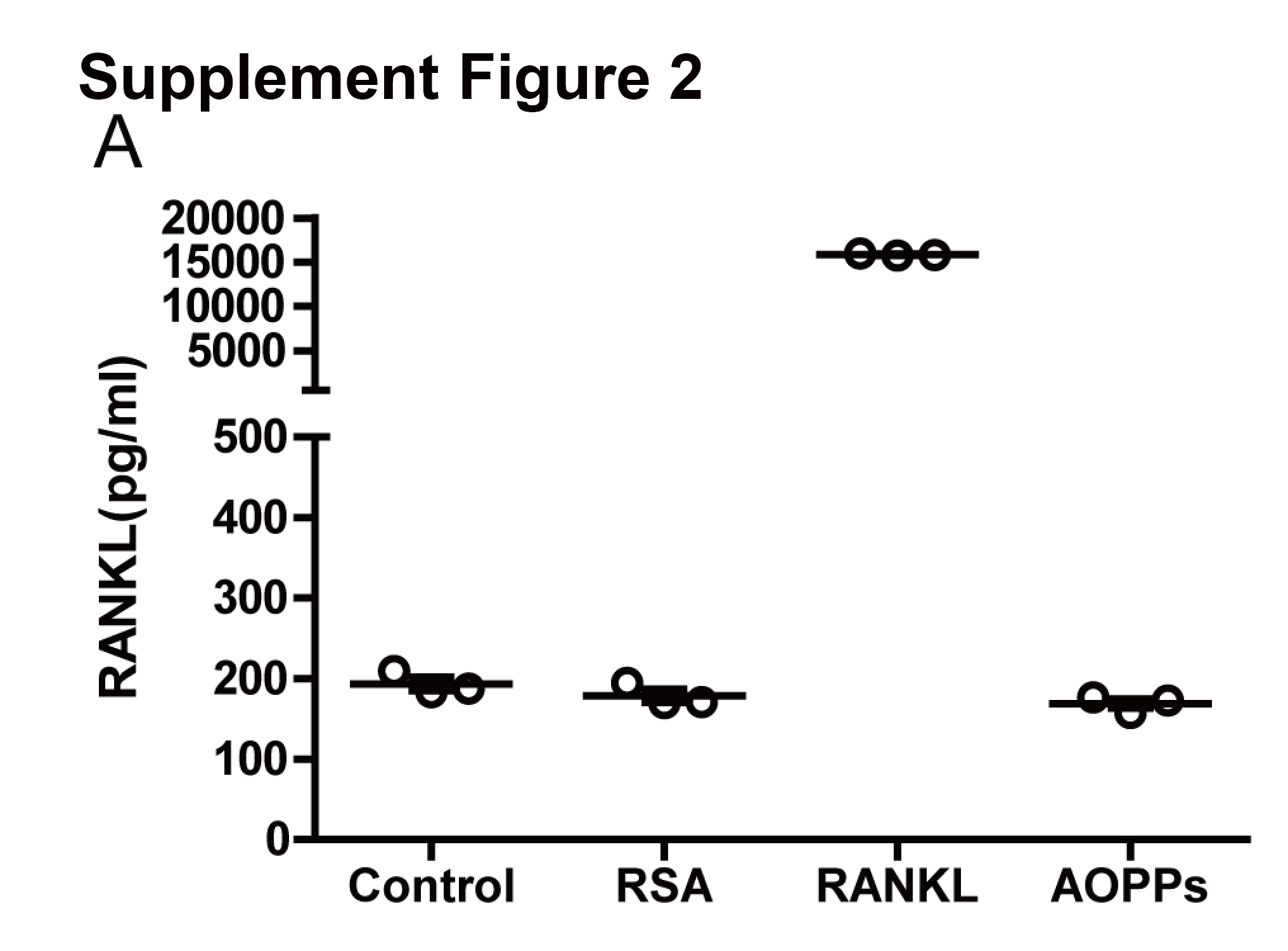

Supplement: Supplementary file 3 — Supplement figure 2 [file 41419_2021_4441_MOESM3_ESM.png]

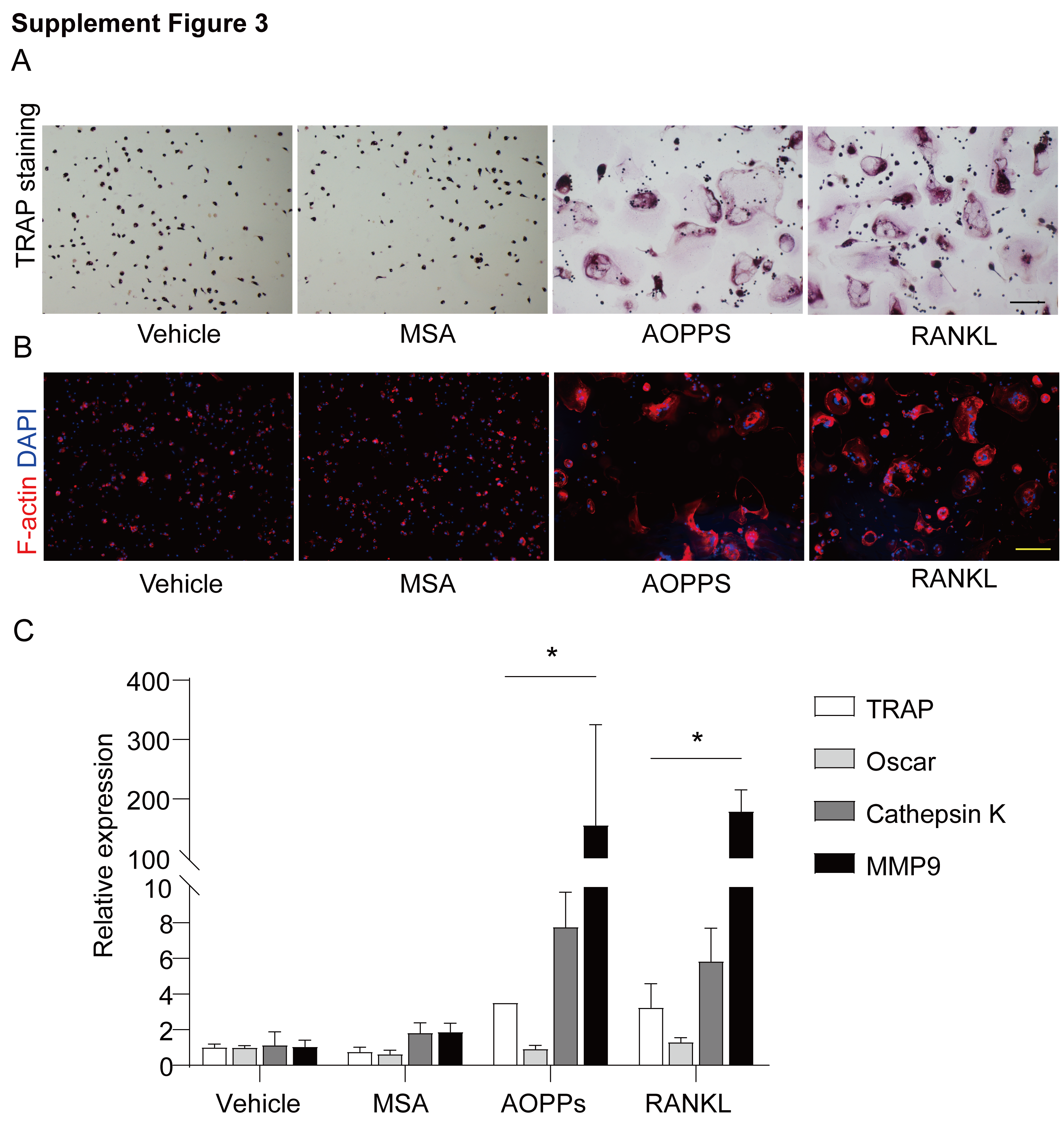

Supplement: Supplementary file 4 — Supplemental figure3 [file 41419_2021_4441_MOESM4_ESM.png]

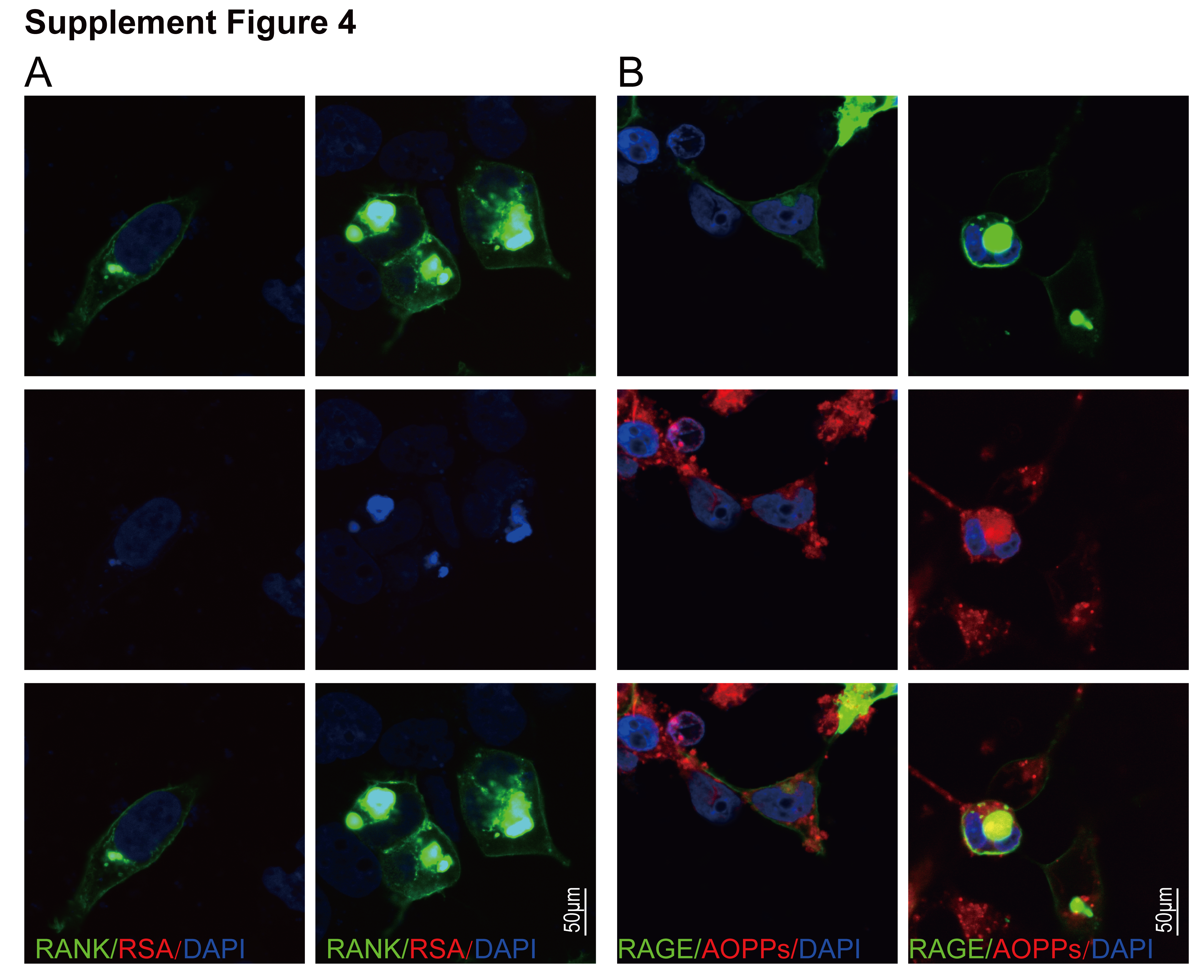

Supplement: Supplementary file 5 — Supplemental figure4 [file 41419_2021_4441_MOESM5_ESM.png]
