# Supplementary material for: Age-related accumulation of advanced oxidation protein products promotes osteoclastogenesis through disruption of redox homeostasis
Source: Cell Death Dis. 2021 Dec 14;12(12):1160. doi: 10.1038/s41419-021-04441-w (PMC8671415; doi:10.1038/s41419-021-04441-w)
Supplement: Supplementary file 6 — Supplement tabel 1 [file 41419_2021_4441_MOESM6_ESM.docx]

**Supplement table 1. Primers for RT-qPCR**

| Gene |  |  |
| --- | --- | --- |
| GAPDH-Forward | 5’-GGCACAGTCAAGGCTGAGAATG-3’ |  |
| GAPDH-Reverse | 5’-ATGGTGGTGAAGACGCCAGTA-3’ |  |
| TRAP- Forward | 5’-TGGCAATGTCTCGGCACAA-3’ |  |
| TRAP- Reverse | 5’-AGCATCACGGTGTCCAGCATAA-3’ |  |
| MMP9- Forward | 5’-AGCCGGGAACGTATCTGGA-3’ |  |
| MMP9- Reverse | 5’-TGGAAACTCACACGCCAGAAG-3’ | |
| Cathepsin- Forward | 5’-CGGCTATATGACCACTGCCTTC-3’ | |
| Cathepsin- Reverse | 5’-TTTGCCGTGGCGTTATACATACA-3’ | |
| Oscar- Forward | 5’-GCCATGGTCCTGTTGCTGATA-3’ | |
| Oscar- Reverse | 5’-GGTGCACGGCAAATCAAAGTTA-3’ | |
