# Supplementary material for: Age-related accumulation of advanced oxidation protein products promotes osteoclastogenesis through disruption of redox homeostasis
Source: Cell Death Dis. 2021 Dec 14;12(12):1160. doi: 10.1038/s41419-021-04441-w (PMC8671415; doi:10.1038/s41419-021-04441-w)
Supplement: Supplementary file 7 — Title page [file 41419_2021_4441_MOESM7_ESM.docx]

**Title page**

**Tile:** Age-related accumulation of advanced oxidation protein products promotes osteoclastogenesis through disruption of redox homeostasis

**Author:** Jingshen Zhuang^1^, Xuebing Chen^2^, Guixing Cai^1^, Dizheng Wu^1^, Chen Tu^1^, Siyuan Zhu^1^, Yusheng Huang^1^, Ping Xu^1^, Zhaoming Zhong^1*^

**Affiliations:**^1^ Spine Division, Department of Orthopaedics, Nanfang Hospital, Southern Medical University, Guangzhou, China

^2^ School of Forensic Medicine, School of Forensic Medicine, Southern Medical University, Guangzhou, China

^*^**Correspondence**: Zhaoming Zhong, Division of Spine Surgery, Department of Orthopaedics, Nanfang Hospital, Southern Medical University, 1838 North Guangzhou Ave, Guangzhou 510515, P.R. China; E-mail: [zhongzm@smu.edu.cn](mailto:zhongzm@smu.edu.cn); tel: +86-020 62787190

**Running title**: AOPPs accumulation promotes osteoclastogensis

**Highlight:**

1. AOPPs accumulation promotes osteoclastogensis.

2. RANK and RAGE dependent redox sensitive signaling were involved in AOPPs- induced osteoclastogensis.

3. AOPPs accumulation played an important role in the development of age-related bone loss.

**Disclosure of Conflicts of Interest:** Authors declare no conflict of interest.
