# Supplementary material for: Age-related accumulation of advanced oxidation protein products promotes osteoclastogenesis through disruption of redox homeostasis
Source: Cell Death Dis. 2021 Dec 14;12(12):1160. doi: 10.1038/s41419-021-04441-w (PMC8671415; doi:10.1038/s41419-021-04441-w)
Supplement: Supplementary file 8 — Author Contribution Form [file 41419_2021_4441_MOESM8_ESM.pdf]

DECLARATION OF CONTRIBUTIONS TO ARTICLE

ADMC

Manuscript Number: CDDIS-21-2699

Journal Name: *Cell Death & Disease* (the 'Journal')

Proposed Title of the Contribution:

Age-related accumulation of advanced oxidation protein products promotes osteoclastogenesis through disruption of redox homeostasis (the 'Contribution)

Author(s):

Jingshen Zhuang, Xuebing Chen, Guixing Cai, Dizheng Wu, Chen Tu, Siyuan Zhu, Yusheng Huang, Ping Xu, Zhaoming Zhong (the 'Authors')

For all *CDDis* articles, each person named as an author in the published version must be able to show he or she has contributed substantially to the article.

Authorship credit should be based on 1) substantial contributions to conception and design, acquisition of data, or analysis and interpretation of data; 2) drafting the article or revising it critically for important intellectual content; and 3) final approval of the version to be published. Authors should meet conditions 1, 2 and 3.

Any person who cannot be shown to have made a substantial contribution to the article cannot be listed as an author in the final version. The name of any person who is deemed to have made a minor contribution can, however, appear in the Acknowledgments section of the article.

Please complete the table below to indicate the contributions of all named authors to the manuscript.

Author Full Name:	Specification of Contribution to the Manuscript:
Jingshen Zhuang	Designed the study, performed the major work of this study, prepared the manuscript for publication.
Xuebing Chen	Perform some experiments and analysis some data.
Guixing Cai	Perform some experiments and analysis some data.
Dizheng Wu	Perform some experiments and analysis some data.
Chen Tu	Perform some experiments and analysis some data.
Siyuan Zhu	Provided technical support.
Yusheng Huang	Perform some experiments and analysis some data.
Ping Xu	Perform some experiments and analysis some data.
Zhaoming Zhong	Designed the study, provided technical support, prepared the manuscript for publication.

Please complete the table below to indicate the contributions of all named authors to the figures.

Figure 1:

Zhaoming Zhong initiated the study. Zhaoming Zhong and Jingshen Zhuang designed the paper. Xuebing chen and Guixing Cai generated the aged rats, collect the tissue and ELISA data,Jingshen Zhuang generated the TRAP staining, Dizheng Wu and Chen Tu generated the ELISA data. Jingshen Zhuang and Zhaoming Zhong aseembled the figure.

Figure 2:

Jingshen Zhuang generated the TRAP staining data, F-actin staining data and electron microscopy data, Xuebing Chen prepared the WB data and Siyuan Zhu provided technical support, Guixing Cai generated the Qpcr data. Jingshen Zhuang and Zhaoming Zhong aseembled the figure.

Figure 3:

Jingshen Zhuang generated the SPR binding-affinity measurement, IF, IP, and FACS, Siyuan Zhu provided the above technical support. Jingshen Zhuang and Zhaoming Zhong aseembled the figure.

Figure 4:

JIngshen Zhuang generated tht IF and WB data, Guixing Cai generated the intracellular ROS production data.Jingshen Zhuang and Zhaoming Zhong aseembled the figure.

Figure 5:

Jingshen Zhuang, Dizheng Wu and Chen Tu generated WB data. Jingshen Zhuang and Zhaoming Zhong aseembled the figure.

Figure 6:

Jingshen Zhuang generated the TRAP staining data, F-actin staining data and electron microscopy data, Xuebing Chen prepared the WB data and Siyuan Zhu provided technical support, Guixing Cai generated the Qpcr data. Jingshen Zhuang and Zhaoming Zhong aseembled the figure.

Signed for and on behalf of the Author(s):	Print Name:	Date:
Zhaoming Zhong	Zhaoming Zhong	2021.07.15
